# Foreign Summary

**Published:** 1840-04-18

**Authors:** 


### FOREIGN SUMMARY.

### PHILIPS' LECTURE ON THE PRINCIPLE AND PRACTICE OF SURGERY.—NO. I.

#### SCROFULA.

*Antiquity of the Disease—Characteristics—Various Forms—Similarity to Tubercular Phthisis—Age at which it most frequently occurs—Countries where most prevalent—Causes of the Disease—Scrofula not generally a consequence of Hereditary Transmission—Is Scrofula contagious?—Communicability of the Disease to a Child through the Milk of a Nurse—Influence of Food, Filth, and Clothing.*

To undertake to fix an epoch for the first ravages caused and produced by scrofula, and to seek in the earlier periods of the world's history, traces and vestiges of this formidable disease, would be to occupy oneself with objects better adapted to amuse curiosity than to further the progress of science. The history of the ills to which flesh is heir, cannot, in fact, be truly useful or interesting to the medical man, unless it instruct him upon the causes, develope the progress, mark the changes, and indicate the remedies applicable to the particular disease. Although we may trace the existence of scrofula among the earliest historical monuments—although we could show it to have been known to the Phœnicians, the Greeks, or the Egyptians—though we find in the works of the predecessors of Hippocrates, data which are evidence of its antiquity—if the assemblage and combination of ideas, opinions, changes, disputes, and successes, do not assist us in forming a fair opinion of the disease, they bring no real advantage, and our investigations will have been useless.

That little exact information can be obtained by consulting such works in the study of this disease, appears evident when we look at the statement of Faure, made in 1752:—"It would (says he) certainly be too long, and not instructive, to consult a great number of authors who have written on scrofula, either generally or *ex professo.* It is sufficient to say that almost all modern authors, and all practitioners of the present day, admit the cause to be an inspissation of that lymph, of which Thomas Bartholin discovered the vessels in 1652."

The ideas current in the days of Faure, have given place to others; but our knowledge of the immediate or exciting cause of scrofula is, at present, little, if at all, more advanced than in his time.

*Definition.*—By scrofula, I understand a disease characterized by the deposition in the greater portion of the tissues of the body, but principally in the lymphatic ganglia, of a product presenting the following physical characters:—If we cut into the tissue of a lymphatic ganglion affected by scrofula, it presents a tolerably dense, grayish substance, interspersed through which, in masses varying in bulk, we find a product of a yellowish white colour, and very much of the consistency of new cheese. Left to themselves, in this state, these tumours communicate a certain irritation to the surrounding tissues, pain is felt, the integument is thinned, ulceration takes place; at first a sero-purulent fluid is evacuated. This is succeeded by cheesy, pultaceous matter. When the whole of this matter is evacuated, the ulcer may heal.

*Forms.*—Scrofula is also presented in the form of cutaneous eruptions, particularly about the head. It also affects particularly certain organs. That species of ophthalmia known as strumous is one of the most frequent, and often one of the most obstinate of the affections of the conjunctiva. Children suffering from this disease awake in the morning with the eyelids glued together; sometimes it is very difficult to separate them; they show great aversion to light. Sometimes the disease proceeds to disorganization of the cornea. Ulcers of a like nature in other parts of the body are frequently developed; but in those cases admitted to be scrofulous, the peculiar deposition which we have considered is usually wanting. In fact these affections are held to be scrofulous because they are commonly coexistent with glandular disease, and because they present many of the characters by which the more obvious forms of scrofula are marked. When the disease affects the articulations, the scrofulous deposition is found there, so also is it in scrofulous caries, in rickets, in the mesenteric disease termed tabes mesenterica. Therefore we are accustomed to regard these affections as scrofulous. Frequently it affects a complex fibrous tissue; for instance, the wrist-joints, the sacro-iliac symphysis. From this results that variety of the disease termed white swelling. Rarely does this end in resolution; suppuration supervenes, and caries of the articular extremities succeeds. Often the ligaments of the knee, the foot, the elbow, are the seat of scrofulous inflammation. If we examine a tumour of this kind, we see the fibrous tissues congested, the bistoury gets through it with difficulty, the areola of their tissues are enlarged; they contain a viscid, yellowish, gelatiniform fluid.—Lassus compared the section of these tissues to that of a lemon. The articular cartilages, which, according to some anatomists, are inorganic, and which others regard as possessing a certain amount of organization, present traces of a similar affection. The articulations, where we find fibro-cartilages, present analogous alterations. The bones are very frequently the seat of a similar affection, especially the spongy bones. If we examine a bone which has been for some time thus affected—if we saw it, the vessels are more developed, and the cells contain a yellowish fatty matter. This condition usually proceeds to caries, or the periosteum is inflamed, detached, and necrosis is developed.

*Similarity to tubercular matter.*—In investigating the nature of this disease, it is necessary to inquire what similarity it bears to tubercular phthisis—what resemblance scrofulous bears to tuberculous matter. Men of great eminence have regarded these as identical products. My own opinion is, that this identity remains to be proved; that the question is yet undecided.—My reasons for doubting their identity, are as follows:—Scrofulous matter, when deposited in lymphatic ganglia, is infiltrated through the interstices of their tissue, like water in a sponge. A consequence of this arrangement is, that a scrofulous tumour is not completely emptied when the parietes are ulcerated, but scrofulous matter continues to be evacuated until the whole glandular structure is broken down; and it is worthy of remark, that when the cicatrization is completed, not a vestige of the diseased gland can usually be discovered. Tuberculous matter, on the contrary, except in a small, comparative, number of cases, whether deposited in the lung, the liver, the spleen, the kidney, the testicle, the brain, is not an infiltration through the tissues of the organs, but a deposition at distinct points. In all the scrofulous tumours I have examined, the softening of the scrofulous matter succeeded to a surrounding suppuration. In the case of tubercles, the softening usually precedes the phenomenon. No doubt, in both cases, the abnormal products is the focus of irritation.

*Period of life.*—By far the larger portion of the cases of scrofula are presented before the age of puberty. The great majority of cases of tubercular phthisis are presented after 18. Of seventeen cases of scrofula where life had been destroyed by the exhaustion attendant upon profuse discharges from ulcerated surfaces and scrofulous caries, only one presented considerable tubercular deposition in the lungs, and nine presented scarcely a trace of it. Of eighty-six cases of tubercular phthisis, only one presented any cicatrix or other evidence in the neck, the axilla, or the groin, of having suffered from disease in the lymphatic ganglia in these several regions. Louis' experience is somewhat different: of 358 cases of tubercular disease of the lung, examined by him, 30 were found to present more or less traces of the deposition of similar matter in the lymphatic ganglia. But even if we assume this to be an average, considering the frequency of both diseases, it is not too much to presume that they were simple coincidences. Again, if the perfect analogy of anatomical characters between these two products were admitted, we might say that the true nature of a disease does not solely consist in the transformation or degeneration of a tissue, but in the anatomical element which affects it—in the nature of the causes which determine it—in the mode of its transmission—the manner of its invasion—the order of succession of the symptoms which constitute it—in the effects of the treatment opposed to it.

*Where prevalent.*—It is also true that the countries other than our own, where scrofula is most rife—Holland, Dauphiny, the Valais, the Vivarais, and certain portions of Brittany, present a smaller number of cases of phthisis than other countries where scrofula is more rarely seen. In the whole of the Oceanic region extending from La Teste to Mimigan, this remarkable fact obtains—the coincidence of extreme frequency of scrofula with excessive rarity of tubercular phthisis.

These are differences which I cannot reconcile; although, therefore, I do not admit absolute identity between these two products, I do not absolutely deny it; I only mention that, at present, it remains to be proved.

*Causes.*—It is said there is a scrofulous diathesis. If, by a diathesis, it be intended to convey a condition of the economy in which the constitution is simply disposed to, but as yet unaffected by, the particular disease, I do not believe in it. I think that what is usually described as a diathesis, is a first symptom, or series of symptoms, of the infection of the system by a disease which will presently show the local characters of the same affection. If this period could be well made out—if it were ascertained that during this period no local deposition of the scrofulous product had occurred—then, no doubt, it would be a matter of great importance to employ such means as would appear to be capable of averting the impending evil. But if this disposition could be removed, science would be very jealous of admitting as facts what two men might be disposed to agree upon—the existence in a particular case of this particular diathesis.

In the second period no such difficulties are experienced; the disease appears with its characteristic symptoms, the organization is modified, the functions are deranged, and the disease is unmasked. The first and commonest system is the tumefaction of the lymphatic ganglia. At first they are small, moveable under the finger, elastic, without pain or change of colour in the skin. The lateral and sometimes the posterior parts of the neck are the first to feel the influence of the disease; therefore, the early examination of these parts is of much importance in establishing the diagnosis. At a little later period the axillary glands may be affected; sometimes those of the groin, or of other parts of the body. Gradually they increase in size and consistency; the ambient structures are affected, and then the mobility is no longer possessed. It is rarely that a single gland is affected; usually, several are almost simultaneously implicated. As they increase in bulk, they approach each other, are confounded, and often a large tumour or chain of tumours (scrophulæ concatenatæ) is the consequence. I have known such a chain to extend from the neck to the mesentery. But there is a peculiarity in these tumours, which suffices, in those persons accustomed to look at them, to distinguish them from all others: it is a certain degree of elasticity which it would be very difficult to describe. They may remain in this state for many years, but their existence is very inconstant; they will even disappear for a time. Usually, however, they make progress, become harder and less moveable, the skin over them gets red, a pain is felt towards the centre of the gland, inflammation proceeds slowly, and suppuration is established; the skin ulcerates, and a thin serous pus is discharged.

There are certain characters impressed upon those persons who are to become the subjects of the ravages of scrofula, which I shall now notice. You must, however, bear in mind, that although this be true in most cases, yet that the conjunction of all the characteristics is not always sufficient to justify us in concluding that the person is scrofulous. Commonly scrofulous children are remarkable for the size of their heads—for a tendency to eruptions, or scurf on the scalp—the intellectual faculties are often greatly developed—for a tumefaction of the free borders of the eyelids—an extreme susceptibility to the impression of light. Schmucker has pointed out the length of the eyelashes as another character; a flattening of the root of the nose, an excessive volume of the lips; in some, the cheek-bones are high and broad. Chaussier attached much importance to the examination of the teeth, believing that the greater number of children disposed to scrofula have bad first teeth. The neck certainly presents two opposite conditions, either it is thin and elongated, or it is short and thick, so that the head seems to be between the shoulders. Very commonly you find in scrofulous patients a vicious conformation of the chest; the thorax is narrow, and arched in front—pigeon-breast, as it is termed; the shoulders are raised: this projection forwards of the chest, renders more sensible another characteristic—the large abdomen. In the greater number of patients threatened with scrofula, the digestive functions are irregularly performed, the appetite is depraved or lost; frequently they suffer from diarrhœa; the skin is usually of a rosy white, fine, transparent, with the cutaneous veins very apparent. Some authors, wishing to find in the colour of the hair a distinguishing sign, have laid down as a rule, that scrofulous patients have blond hair. This proposition is much too general. Observation demonstrates, as I shall presently show, that we meet a larger number of the shades of dark than of blond in this disease. There are certain distinctive characters presented by the extremities: usually they are thin, without the ordinary quantity of muscular power; but the articulations are large, and this is especially apparent at the knee and the elbow. Sometimes this apparent enlargement is delusive, and is produced by the want of natural fulness in the limb; at other times disease has commenced in the articulations, and tumefactions exist there, affecting, principally, the fibrous tissues. Again I repeat, that each of the appearances I have enumerated may exist without a scrofulous constitution; but their conjunction is most important, and calculated to produce a conviction of the existence of the disease we are considering.

Of all the infirmities which afflict the human species, scrofula is certainly one of the most tedious and most difficult to cure. It belongs to the class of diseases termed constitutional; that is, a disease so identified with the economy, that a considerable change or impression must be made upon the system generally, before we can destroy this condition.—Nature seems to have much more power than art in bringing about such a revolution. It is not rare that we see the strumous constitution losing its intensity, and completely effaced, under the influence of those changes brought about at critical periods of life.

Considered in its ordinary limits, scrofula is a disease proper to an early period of life, and also a very common one; it is not of itself mortal, but may become so by affecting organs essential to life. It may terminate in many ways, either by complete resolution, leaving no trace, which unfortunately is rare. Suppuration is the ordinary termination. It varies singularly in its duration. When it is preceded by acute and painful inflammation, it is rapidly brought about; pus does not then present the usual characters, of scrofulous suppuration. When, on the contrary, the inflammation is slow, the pus is thin and serous; it has not that white creamy aspect which we see in healthy pus; it is not thick, but whey-like. These tumours ordinarily contain "tuberculous" matter, consistent or softened. When scrofula affects bones, it has analogous terminations. Most commonly its progress is slow; this is partly owing to the nature of the affected tissues, but it may terminate by resolution. Often the fibrous tissues which surround an articulation, form large masses; "tuberculous" matter is deposited in their interstices, and the joint acquires sometimes a very large size.

There are certain diseases common to our climate, the ravages of which are unquestionably increasing in frequency, if not in intensity; and of these, scrofula is one. Our power over this disease, when once developed, is comparatively inconsiderable. By that I do not mean that we are not frequently able to combat with success glandular depositions, but the disease is constitutional, and this it is difficult to modify so as to eradicate the disposition to new deposits when old ones are removed. We cannot, therefore, reasonably hope to lessen its ravages until we can exert some restraint over those causes under the influence of which it would seem to be developed; to them consequently much attention should be devoted, for the purpose of determining the more efficient of these causes, and the extent of our power to exercise over them any control.

Among the causes of this disease numerous agents have been ranged; of these the principal are hereditary transmission, lymphatic temperament, contagion, syphilis, food, filth, clothing, vitiated air consequent upon imperfect ventilation. Now with respect to these so-called causes, no doubt one and all of them may be so mixed up with particular cases, as to render it difficult to prove that they may not have stood in the relation of cause and effect; but such apparent relation in particular cases will be dissipated, if I show that, in a very large majority of cases, one or more of these causes has been absolutely wanting.

*Hereditary transmission.*—If we assume tubercular and scrofulous matter to be identical, I do not hold that the specimens referred to in different museums, where tubercles were developed during intra-uterine life, support this conclusion, because it is not shown that in these cases the parents presented any similar condition, and because unquestionably diseases may be developed during fœtal life which the parents did not present. It may be said, and no doubt truly, that "hereditary" diseases are not necessarily manifested at the moment of birth; but I think that it is equally true, that there is a great want of cohesion in the idea generally current with regard to hereditary diseases. Cullen states that he knew a family the father of which was tainted with scrofula: all the children who resembled him were scrofulous, whilst those who resembled the mother were exempt. It is necessary to bear in mind that Cullen was strongly preoccupied in favour of hereditary transmission in this and other diseases. In the affected families which I have examined, the father was affected only once, whilst the mother had suffered in eight cases; and my own observations in this and other hereditary transmissable diseases lead me to the conclusion, that the mother is a much more important agent in this mode of propagation than the father. Again, with respect to this influence, those who, like Cullen, believe that it is almost always hereditary, or like Lemasson Delalande, who stoutly maintained that it could never be acquired, find themselves frequently involved in serious difficulties, especially when it is impossible to pronounce for the existence of similar disease in the parents; but there is no difficulty so great as that it may not be overleaped; and this difficulty is dissipated by passing back through one, two, three, or any other number of generations, until we find some ancestor in whom the disease had existed; and when we consider how commonly, unhappily, this disease is seen in our own land, it would be unfortunate, indeed, if it were necessary to pass through more than two or three generations before we arrived at some miserable sufferer from its ravages. Kortum clearly set forth the cause of this error when he said, "Fuere è recentionibus varii qui similes progeniei et parentum morbos à simili diæta et vitæ genere potissimum repeterent."

I will now proceed to offer direct evidence in favour of the opinion I entertain, that scrofula is not, ordinarily, a consequence of hereditary transmission. I do not propose to seek to establish that such transmission is impossible. Eighty-three children, presenting unequivocal signs of scrofula, in various forms, and living in the parish of St. Marylebone, were found to be the issue of fifty-eight marriages. Of the hundred and sixteen parents, eighteen were either dead or missing; and of the remaining ninety-eight, nine only presented any marks of scrofulous affection. In none of these cases were both parents affected. The children proceeding from these nine families were in number thirty-nine, and of these, only eleven presented any of the ordinary forms of scrofula. Of these eleven, three were found in one family, and one in each of the remaining eight. This evidence appears to me strongly to favour the conclusion, that if hereditary transmission have any influence in the production of this disease—that if the cause rest upon the parent at all, of entailing involuntarily upon the offspring the disease we are considering—it does not exist to the extent which is commonly supposed; and I cannot admit it to be proved at all.

Of the families indiscriminately taken, in which scrofula was found to affect one or more of the children, only two in fifteen presented the disease either in the father or the mother; and these families actually presented a smaller proportion of cases than those families in which neither parents presented any mark of the disease.

The *lymphatic temperament*, so much insisted on as a cause of this disease, does not, I apprehend, cause the disease at all; in fact, of the eighty-three cases already referred to, forty-six presented dark chestnut or black hair, dark complexion, dark eyes, active and spare habits; whilst of the remaining thirty-seven much difference of opinion existed as to the class to which they belonged—at least ten of them should be excluded from the temperament termed lymphatic. Therefore, I would maintain that the opinion of Richerand, that the lymphatic temperament exaggerated, constitutes scrofula, is incorrect; and the opinion I entertain on the subject is, that the particular constitution which it is said especially predisposes to scrofula, is nothing else than a constitution upon which scrofula has already seized, and impressed certain marked characters—such as a fine transparent flabby skin, a large face, thick lips, with a great tendency to crack, frequent eruptions on the scalp, stoutness conjoined with feebleness, more imagination than physical power. I believe, then, that the lymphatic temperament in no way predisposes to this disease, but if other causes excite its production, I admit that the lymphatic temperament would offer less resistance to its development than any of the others.

The question, whether or not scrofula be capable of communication by *contagion*, is one of so much importance to the happiness of families, that it might naturally be expected that considerable attention would be devoted to the subject, for the purpose of determining whether separation or isolation which is often so distressing, be a precaution which the heads of families could not neglect with impunity.

Happily, at this moment, the commonly received opinion is, that the disease is not contagious; and I am not about to state any thing which can throw doubt on such a desirable conviction, but I am bound to lay before you the evidence, or at least such of it as is material for you to know, upon which this conviction rests. It is true that in the last century, the question of contagion, as a quality of this disease, was submitted to the Faculty of Medicine of Paris, by the Parliament; it is equally true that an affirmative answer was returned; Dulaurens stating, "Contagiosum esse multi experiunter." Of course, so long as the disease was supposed to be the result of a particular virus introduced into the economy, the probability of contagion could be with difficulty denied; but in the present day, when that opinion is no longer tenable, the advocates of contagion are dropping off. In the Hôspital des Enfants, at Paris, where, commonly, from a hundred to a hundred and fifty beds are occupied by scrofulous patients, exhibiting the disease in every stage and form, no facts have been observed to warrant this opinion. In a school in my own neighbourhood where the disease is very commonly seen, the sufferer from scrofula, unless confined to bed, mixes indiscriminately with those who are healthy—at meals, at play, and at night they occupy the same dormitories; but no circumstances have ever occurred to warrant a suspicion of contagion. In families, we find two brothers, or two sisters, sleeping together, one suffering from this disease, the other free from it, but no communication. I therefore unhesitatingly say with Kortum:—"Quotidie occurrunt exempla ubi sani infantes cum scrophulis arcto et ipsius lecti consortio fruuntur, nec tatem ipsis morbus communicatur." But then it is maintained, that in families we very frequently see the disease developed in one child after another, until a whole family have been infected, and that here contagion must be admitted. At this moment I know a family in which the disease has occurred in an aggravated form in every child but one of a large family; but in none of them is it manifested before the age of seven: the one who has escaped was removed from home at eight: the father and mother are free from it; but every child, save one, has acquired the disease—not by contagion, for they would have suffered earlier in that case, in consequence of being much more together than at any subsequent period—they acquire it under the influence of circumstances to which I shall presently allude. They resist it until they are pulled down by the irritation attendant upon the second dentition.

With respect to direct experiment, many objections may apply to it; certain diseases may be caused by respiration; certain others by the direct and simple contact of a virus; others by inoculation. In the disease under consideration, upon which test should we rely? what fluid or solid contains the germ of the disease? Hébréard has inoculated dogs with scrofulous pus without success; Lepelletier has repeated the experiment upon Guinea pigs with a like result; Kortum has rubbed the neck of a child with pus furnished by a scrofulous ulcer—he has even made a wound behind the mastoid process for the purpose of inoculating a child with similar pus, but without exciting the disease. But when should this pus be taken?—Sœmmering believed that the experiment would succeed if the ulcer presented the characters of this disease in considerable intensity, but it is a mere matter of opinion. A colleague of Lepelletier, at the same time that he vaccinated many other children, inserted pus proceeding from scrofulous ulcers. The vaccine virus manifested itself in the ordinary way, but scrofula was not developed. I can conceive no justification which this person could offer for this wanton outrage. Lepelletier felt this, and made himself the subject of experiments; he inserted similar pus under the integument in various parts of his body, but no symptom of scrofula was manifested. Again, to test the opinion of those who maintain that the contagion of this disease resides in the cutaneous transpiration, he inserted under the cuticle, at several points, the fluid taken from a blister applied upon the body of a scrofulous sufferer; a little suppuration occurred at one puncture, but on the fourth day it was entirely dissipated. Mr. Goodlad has performed similar experiments, and with similar results. To the case of Rowley, in which, he says, the inoculation of small pox produced scrofulous tumours in the neck, I attach no importance, because it does not appear that the virus was taken from a scrofulous person, and because small-pox appears frequently to excite the development of scrofula. De Haen maintains that scrofula more commonly succeeds to inoculated than natural small-pox. Cullen supports an opposite opinion.

To my mind these experiments carry no conviction either way. And if experiment be capable of determining the question, it is yet to be made. If I were to perform any experiments on the subject, I should not employ the pus proceeding from a scrofulous abscess.—And for this reason; there seems to be a period when the ulceration which marks certain contagious diseases does not furnish a pus capable of propagating that disease; I would, therefore, prefer inserting under the integuments, the tubercle-like matter, which is the marked pathological characteristic of the disease in question.

I do not think it necessary to occupy your time long with the question of the communicability of the disease to a child through the milk of a nurse, because I can throw no light upon the matter. Bordeu thought it was impossible to deny it. How, says he, can you refuse to admit, that a virus so intimately mixed with solids and fluids should not be communicated by means of the milk to a sucking child? First, it is necessary to show that a virus exists; and next that it must necessarily be contained in the milk. Syphilis pervades the system pretty completely, and yet I am not aware of any case in which the disease has been communicated to the child solely by means of the milk of the affected person. Direct experiment here is difficult; we do not choose a scrofulous nurse to suckle a child; and if the mother be the scrofulous nurse, we have no more right to believe that a virus has been contained in the milk than that the disease was hereditary; for then we should have to deny the influence of other agents, which we shall speedily consider. So much, however, is certain, that if we admit—which I have not done—a similarity in nature amounting almost, if not altogether, to an identity between scrofula and tubercular disease, we may then support ourselves by this fact, that in those cows which suffer from tubercular deposits, the milk presents, as was shown by Labillardière, seven times more phosphate of lime than is found in the milk of a healthy cow. If we admit for the moment, that the milk of a woman suffering from scrofula undergoes a similar change, we have yet to prove that such a change in milk is capable of producing scrofula. Of one thing I have little doubt, that the milk of a person so suffering is less fitted for the purposes of nutrition than that of a healthy woman; but it is not proved that defficient nutrition alone is the cause of scrofula.

*Syphilis.*—If we take as a fact—the truth of which is generally admitted—that syphilis was unknown in Europe until the return of Columbus from his first voyage to the shores of the New World, it must, I apprehend, be also conceded, that scrofula was not originally "degenerated syphilis." I am, therefore, at a loss to understand any sufficient ground why men of considerable reputation have so stoutly maintained this hypothesis. These authors, it is true, maintain that there is the greatest similarity between the two diseases; that both produce ulcers of the skin and caries; that both affect the lymphatic glands, which become tumid; that both are cured by similar means. To me, these positions appear preposterous. Take first the glands; in syphilis, it is those of the groin or thigh which are usually affected; in scrofula, those of the neck: in syphilis, caries usually affects the head or neck; in scrofula, the extremities, and especially their points of articulation, principally suffer. Mercury is the remedy in syphilis; mercury similarly administered, is a most baneful method of treating scrofula. There are other reasons which may be brought to bear against this hypothesis; that in many situations, syphilis is extremely common—scrofula, very uncommon. Take Palermo, for instance, where syphilis is probably more rife than in any part of Europe, scrofula is comparatively rare. Therefore, even if we admitted that syphilis was known in Europe from very early times, it is still far from being proved that it is the cause of scrofula; but I apprehend it to be clearly made out, that scrofula has existed in Europe for centuries long anterior to syphilis. I am aware that those who maintain the hypothesis we are considering, deny this early existence of scrofula, and urge in support of their plea, that struma and scrofula are different diseases. But any one who reads, attentively, Celcus, Guy de Chauliac, or Roger of Parma, can urge no valid objections in support of this opinion. Again, if we take the power conceived to be possessed by kings, of curing this disease by the royal touch, we may follow this disease from the days of Clovis, in the fifth century, up to a very recent period. In the absence, therefore, of better evidence, tradition would come in support of the identity. Clovis was supposed to have derived this power, as all other kings were conceived to have acquired it, by inunction. I know it is believed by some persons that syphilis existed in Europe long before the days of Columbus, and that the Book of Leveticus has been appealed to in support of this view of the subject, as well as the work of William of Salicetus, whose description seems to be more specific, and which bears date 1280; but I know, also, that the best authorities are opposed to this view of the matter.

I am not prepared to assent to the generally received *opinion*, that *food*, *filth*, and *clothing*. have any very direct influence in favour of, or in opposition to the development of scrofula; neither am I disposed to admit the correctness of the statement, that of the cases of scrofula which come under consideration, a very large proportional majority are found to afflict the poor. I say proportional, to guard myself from misinterpretation, because, as the poor constitute a majority of the people in all lands, there can be no doubt that they furnish a majority of the cases of scrofula. All that I wish to convey is, that this affliction does not fall much more heavily, in a given number of cases, upon the poor, than upon those whom Providence has placed in a better condition. My reasons for believing that the three circumstances above alluded to have no great influence in producing this disease are as follows. A friend has furnished me with the following results obtained from one parish in Wiltshire:—"There are in this parish 49 families, the heads of which earn seven, eight, or nine shillings per week. The number of children in these families amounts to 163; they have, many of them, scarcely rags to cover them; they scarcely get any animal food, and live principally on what would seem to be an insufficient quantity of coarse bread, potatoes, and some butter milk. Of these children only three presented any of the usual symptoms of scrofula." In four courts in the parish of St. Marylebone, I have found 93 families, containing 201 children, the greater number running about, some engaged as errand boys, and so on; very few with shoes or stockings; most of them with clothing insufficient to cover them; scarcely any of them with enough to protect them from the cold; fed upon pretty good bread, potatoes, and an occasional piece of meat—in fact much better fed than the children of the Wiltshire agricultural labourer. Of these children 19 presented manifest signs of scrofula, affecting the glandular system, the eyes, or the bones. Now as far as food, clothing, and probably cleanliness, are concerned, the Marylebone children were at least as well off as those of Wiltshire: whence comes, then, the greater frequency of scrofula in the former? Take the people of Palermo, and who are worse fed or worse clothed than they are? Their food frequently unripe fruit, a little Turkey wheat boiled in water, and probably a little fish; and what people more free from scrofula than they? Scrofula is occasionally endemic at Gottingen, and is attributed to the use of the potatoe as a principal article of food—a speculation which was hazarded by Haller. It is said again, that it is to the *food* that the negroes transplanted from Africa into Europe owe the development of scrofula. To what, then, do the animals introduced from torrid climes to our own owe the developement of scrofula and tubercles? The lion and tiger eat only animal food in a state of nature; they get only animal food here. The monkey eats fruit in his own land; he gets it here. Some other influence than food must, I apprehend, be in active operation to produce scrofula. There can, however, be no reasonable doubt that food of bad quality or deficient in quantity, may prejudice the health—may excite the mesenteric glands to unhealthy action—may, in fact, lay the patient open an unresisting victim to the attacks of scrofula, or any other disease.

A few remarks seem necessary with regard to *water*. It has been stated by many writers that the inhabitants of certain valleys in our own and in other countries owe the frequency of their suffering from this disease to the water which they use (snow water;) but the only difference which has been distinguished between this water and river water is, that it contains a smaller portion of atmospheric air. But then, according to the evidence of Saussure, the mountaineers drink the same water as the inhabitants of the valleys; and among them scrofula is rare. It is true that, at Rheims, there has long existed an opinion that stagnant water produced scrofula, and that, in consequence of this impression, a rich and benevolent canon devoted a part of his riches to the construction of an hydraulic apparatus, by which a stream of water from the Vesle was directed upon the town. It is further stated, that after a few years the number of cases of scrofula diminished. When Desgenettes inspected, in 1806, the Hospital of St. Marcou, specially devoted to scrofula, he ascertained from the registers of this institution that the number of cases had greatly augmented since the water-works had got out of repair. Now these circumstances, which appear so like a true narrative, are contested by Baudelocque, who says in 1777, Laignières, after examining the registers of the same hospital, stated that the number of cases of scrofula had diminished more than half since about thirty years; and that these water-works had only been constructed in 1753, and that therefore the water could have nothing to do with it. Whether or not the water had the effect attributed to it, may still be a question; but I cannot help thinking that Baudelocque's reasoning is hardly satisfactory—in fact, too strained.—About thirty years, may often mean twenty-four; and *since* may often mean *during*. He would rather refer the amelioration to the widening of certain streets, which seem to have been *ordered* in 1755, and to the improvement in sewerage, which occurred between 1732 and 1748. It is not denied that the disease has increased there since the waters of the Vesle are no longer directed upon the town; neither have the wide streets been reduced to their pristine narrowness; nor have the sewers again become uncovered; therefore upon the above data, we think the water advocates have the best of it. But further evidence would be required before we should be authorized to pronounce judgment. Among the information that is required is the state of the poorer classes. New operations of manufacturing industry have, I believe, been introduced, and the population of certain quarters of the town has become much more dense—something like our Spitalfields. The influence of this state of things should be weighed.

If *filth* were a cause, how fearful would be the ravages of the disease! See the German Jews: can any thing be more filthy than their children? And it is true the disease is frequent among them; but take children upon whom every care is lavished, they become the victims of scrofula. Can any thing be more filthy than the people of Palermo? yet the disease is comparatively rare there. Take our own country, the people of which are reputed among the most cleanly in Europe, and the disease is extremely common.

*Atmospherical influences* in the production of this disease, if they have any effect, are inconsiderable. If you deprive vegetables of light, they become bleached or colourless. If you deprive a human being of light, a somewhat similar appearance is produced in his person; but whatever may be said, this state is not scrofula; it may be a state of debility predisposing to any disease.

With respect to *temperature;* it is in those countries which are temperate, that the disease is most commonly seen. Once developed, the transitions from warm to cold are decidedly injurious. In the St. Marylebone Infirmary, the amendment in cases of scrofula, which during the summer and the beginning of autumn has been progressive, is interrupted; and on the approach of winter, they very frequently commence a retrograde march; but my registers do not show any great proportional increase of application for admission or attendance during winter than is observed with regard to most diseases. M. Baudelocque maintains that this is not owing to cold, but to the patients continuing longer in bed, using no exercise, and remaining in a ward where the air undergoes no sensible change from day to day, and where, therefore, a vitiated air is constantly respired: he is still more firmly impressed with a conviction of the correctness of this view by the fact that among private patients this stationary condition or retrograde movement is not observed, and that the action of anti-scrofulous medicines is unchanged. That this is the result of M. Baudelocque's experience I do not doubt, but I fearlessly ask those who have frequent opportunities of observing the disease in our own countries whether their experience is not the converse of this? My own opportunities of observation have been considerable, and certainly the retrograde tendency in scrofulous ulceration during winter is very nearly as remarkable as I have usually seen in the wards of an hospital.

With respect to our own land, it may be interesting to inquire, whether the *humidity* of our climate exercises any particular influence in the development of the disease. Holland is a more humid country than our own, and probably suffers more from scrofula; but then many very dry countries are equally afflicted—many damp ones are nearly exempt. I am, therefore, inclined to believe, that alone, this is insufficient to excite the development of the disease, but the data necessary for determining the question are wanting.

It is believed, by M. Baudelocque, that the "*vitiation of air* consequent upon insufficient ventilation, is the *true*, perhaps the *only* cause of scrofula—that where there are scrofulous people this cause exists—that wherever it exists there shall we find scrofula—and that where it is wanting, scrofula is unknown." I would readily concede to him, that in large towns scrofula is most commonly found in those densely peopled quarters where ventilation is ill performed, but then it should also be borne in mind that there are other causes of disease also in action. No one in our own country will deny that although scrofula is found in the greatest quantity where dense masses are collected in small spaces, that it is also found where houses and rooms are lofty, streets wide, and every care lavished: no one will, I apprehend, deny, that in the houses of the rich and great, in towns and out of towns, this disease is very frequently developed, where the cause in question does not appear to be in operation. He triumphantly refers to Spitalfields in support of his opinion, and compares it with Whitechapel; in the former, says he, the entire population is tainted with scrofula, and a large proportion have crooked spines, are pale, emaciated, and miserable; the young man of twenty looks forty; no aged person is seen unmutilated; crooked backed, round-shouldered, bow legs, and long arms. A man above five feet is a giant. In the latter, says he, which is an adjoining quarter, the houses are better, the occupations of the people are different; they are not heaped into small rooms—and the people are vigorous, "well-built," and good-tempered. From the inquiries I have made, I would incline to the opinion that scrofula is much more common in Spitalfields than Whitechapel, but Baudelocque's preoccupation in favour of his theory, must have been pretty strong, and his information extremely inaccurate, to have enabled him to paint such a picture of the inhabitants of Spitalfields: although I admit, as a fact, that the inhabitants of Spitalfields are greater sufferers from this disease than their neighbours of Whitechapel, yet my data are very insufficient to warrant me in speaking of it as an established fact: it must be borne in mind that many portions of Spitalfields are not so densely populated, nor so completely occupied, as he would suppose, with their looms; and that a good deal of the adjoining portions of Whitechapel are similarly tenanted and similarly occupied with corresponding portions of Spitalfields.

Again, he states, what my own observation convinces me to be perfectly true, that a very large quantity of this disease is developed in persons occupying a somewhat different rank in large towns—small shopkeepers; and with respect to them, we may apply the same reasoning as to the poor weavers—they rarely escape from their houses except for a few hours on Sunday, and not always then. The upper part of their houses is usually let out to lodgers; their only retiring room is often a miserable little room behind the shop, which often also serves as a bed-room. Ventilation is scarcely at all effected, and from day to day very little change takes place in the air of these rooms, which must become extremely vitiated, and very unfit for the purposes of respiration. Adults may and do struggle against such a noxious influence for a long time; but we need only look at a large portion of this class of people, to be assured that their health is seriously undermined—that their power of resisting disease is greatly weakened. With children, however, the case is more serious; for a few hours a day they may be sent to a neighbouring school, but this does not better their situation; from a small room at home, ill ventilated, and with perhaps four or five persons occupying it, they proceed to another room hard by, little if at all larger, and occupied by from twenty to fifty persons. Such is the life they lead, at a time when they are most susceptible to the attacks of disease; and there can be, I apprehend, little doubt, but that the mean duration of life in this class is much shorter than in persons of a similar class, whose avocations do not confine them to the house, or to such small rooms. And after the register of the Enfants Trouvés, and other establishments devoted to children, it results that the ravages of scrofula are more decidedly felt, whilst the children remain in these establishments, and that their health becomes sensibly ameliorated when they are sent to small houses in the country. In the larger receptacles a large number is confined in comparatively small spaces, and comparatively little opportunity for exercise is afforded them. In the country, although the rooms are small, the number occupying them is also small, and their facilities of taking much exercise are almost unlimited.—*London Medical Gazette.*

---

*Consequence of Ligature of the common Iliac Artery, near the Bifurcation of the Aorta.* By Professor Salomon, of Petersburg.—In this case the left common iliac artery was tied nearly a year before the death of the patient, for an aneurism of the external iliac. The cure was deemed perfect; the tumour almost entirely disappeared, and the free use of the limb was restored. After remaining well for ten months, the patient exposed himself to the cold in the open air during a stormy night, with but little clothing on. Rheumatic inflammation of the psoas muscle (*psoite rhumatismale*) was brought on, and though treated by the most energetic antiphlogistic means, suppuration could not be prevented. An abscess formed, and was opened three weeks from the beginning of the symptoms, just below Poupart's ligament. He died shortly after, worn out by the suppuration. Before examining the body, the abdominal aorta was injected. Inspection showed that pus was collected along the psoas, beneath the fascia iliaca, and on the outer side of the femoral vessels. The iliacus internus muscle was as it were dissolved by the ichorous pus, and the internal surface of the ilium was exposed. The abscess had formed outside the peritoneum along the outer portion of the aneurism; but the pus had not passed inwards to the femoral ring; at this part there was a fibrous mass which remained from the internal portion of the aneurism. No fibrous clot was found; it had no doubt been already completely absorbed.

The injection had passed into both legs. It was easy to see by the contracted and firmly adherent part of the left common iliac that it had been tied about half an inch below the bifurcation of the aorta. It was converted into a ligamentous cord throughout its whole length. A little of the injection had passed into the left external iliac through the medium of the left internal iliac artery. The maintenance of the circulation was chiefly effected by the very dilated lumbar arteries, whose branches anastomosed with those of the left circumflexa ilii. The lower extremity was also in a great measure supplied with blood through the free communications between the two internal iliacs. The left femoral artery was injected to within two inches below Poupart's ligament. The common, external, and internal iliacs, on the right side, were considerably dilated; and in the left thigh it was chiefly the ischiatic and obturatrix arteries that had increased in size. *Zeitsch. fur die gesam. Heilk and Gazette Medicale.* Dec. 14th, 1839.

---

*Amputation of the neck of the Uterus,—torsion of the Arteries.* By M. Amussat.—M. Amussat has recently shown, that torsion of the arteries is applicable to operations performed on the uterus.

Madame P., thirty-two years of age, married at the age of twenty-nine; had two children, and aborted once, five months before the commencement of her present illness. Since her accident, she was attacked with metroperitonitis, and continued to lose blood from the vagina, from time to time. A month back, she was examined by a medical man, who discovered the existence of some serious malady, and brought the patient to M. Amussat. The latter, also, having examined the patient, found, that the posterior lip of the uterus was greatly enlarged, and occupied by a fungous ulcer. It was therefore determined to extirpate the disease; and, on the 21st of January last, M. Amussat amputated the neck of the uterus in the following manner:—The patient was sustained by assistants, in the position commonly chosen for lithotomy: two hooks were then fixed in the tumour, being conducted along tbe index finger, and slight traction was employed, but the parts gave way, aud it became necessary to fix them again on the anterior lip of the os tincæ. The neck of the uterus was now brought into view, and almost completely separated by a straight bistoury. An artery, which bled freely, was now twisted, and the section was completed with a curved pair of scissors. Cold injections were thrown up the vagina, but the hæmorrage was very insignificant. Since the time of the operation, the patient continued to do very well, and on the 28th, every prospect of a speedy recovery was entertained.—*Lancet.*

